# RETRACTION: Effect of adding Aloe vera jell on the quality and sensory properties of yogurt

**DOI:** 10.1002/fsn3.4171

**Published:** 2024-05-02

## Abstract

**RETRACTION:** Ali Ikram, Syed Qasim Raza, Farhan Saeed, Muhammad Afzaal, Haroon Munir, Aftab Ahmed, Muhammad Babar Bin Zahid, and Faqir Muhammad Anjum, “Effect of adding Aloe vera jell on the quality and sensory properties of yogurt,” *Food Science & Nutrition*, *9,* (2021): 480–488. https://doi.org/10.1002/fsn3.2017.

The above article, published online on 13 December 2020 in Wiley Online Library (wileyonlinelibrary.com), has been retracted by agreement between the journal's Editor‐in‐Chief, Y. Martin Lo, and Wiley Periodicals, LLC. The retraction has been agreed to given evidence of an insufficient peer review process and the conflict of interest of a reviewer. The editorial office found unambiguous evidence that the manuscript was accepted solely based on compromised and insufficient reviewer reports. This evidence was further investigated and verified by the publisher and the Editor‐in‐Chief. As a result, the conclusions reported in the article are not considered reliable as the article was accepted based on compromised peer review. The authors were notified of the retraction and disagreed with this decision.

**RETRACTION:** Ali Ikram, Syed Qasim Raza, Farhan Saeed, Muhammad Afzaal, Haroon Munir, Aftab Ahmed, Muhammad Babar Bin Zahid, and Faqir Muhammad Anjum, “Effect of adding Aloe vera jell on the quality and sensory properties of yogurt,” *Food Science & Nutrition*, *9,* (2021): 480–488. https://doi.org/10.1002/fsn3.2017.

The above article, published online on 13 December 2020 in Wiley Online Library (wileyonlinelibrary.com), has been retracted by agreement between the journal's Editor‐in‐Chief, Y. Martin Lo, and Wiley Periodicals, LLC. The retraction has been agreed to given evidence of an insufficient peer review process and the conflict of interest of a reviewer. The editorial office found unambiguous evidence that the manuscript was accepted solely based on compromised and insufficient reviewer reports. This evidence was further investigated and verified by the publisher and the Editor‐in‐Chief. As a result, the conclusions reported in the article are not considered reliable as the article was accepted based on compromised peer review. The authors were notified of the retraction and disagreed with this decision.

